# Cardiovascular adverse reactions associated with escitalopram in patients with underlying cardiovascular diseases: a systematic review and meta-analysis

**DOI:** 10.3389/fpsyt.2023.1248397

**Published:** 2023-09-22

**Authors:** Kenichi Shizukawa, Hisashi Narita, Hissei Imai, Hisashi Akiyama, Shuhei Ishikawa, Ryo Sawagashira, Tomoyuki Isoyama, Mariko Nohara, Michiyo Kawamura, Yukari Kono, Takuya Saito, Ichiro Kusumi

**Affiliations:** ^1^Department of Psychiatry and Neurology, Hokkaido University Hospital, Sapporo, Japan; ^2^Department of Health Promotion and Human Behavior, Graduate School of Medicine/School of Public Health, Kyoto University, Kyoto, Japan; ^3^Department of Physiology, Hokkaido University School of Medicine, Sapporo, Japan; ^4^Creative Research Institute, Hokkaido University, Sapporo, Japan; ^5^Medical Sciences Group, Research Support Division, Hokkaido University Library, Sapporo, Japan; ^6^Department of Child and Adolescent Psychiatry, Hokkaido University Hospital, Sapporo, Japan

**Keywords:** escitalopram, cardiovascular disease, major adverse cardiovascular events, systematic review, meta-analysis

## Abstract

**Background:**

Despite the anticipated efficacy of escitalopram in treating depression and anxiety in individuals with preexisting cardiovascular conditions, persistent concerns regarding its adverse effects have emerged. In this systematic review, we aimed to evaluate the cardiovascular safety profile of escitalopram compared with that of placebo in patients with underlying cardiovascular disease.

**Methods:**

We used a predefined search strategy in PubMed, Cochrane Central Register of Controlled Trials, Embase, International Clinical Trials Registry Platform, and ClinicalTrials.gov to identify studies evaluating adverse cardiovascular reactions to escitalopram in patients with underlying cardiovascular disease. Randomized controlled trials (RCTs) that provided results on cardiovascular safety outcomes were included. Two independent reviewers screened the abstracts and full texts of the individual studies. The risk of bias was assessed using version 2 of the Cochrane risk-of-bias tool for randomized trials. The certainty of evidence was assessed using the Grading of Recommendations, Assessment, Development, and Evaluation approach.

**Results:**

The primary outcomes were the frequency of major adverse cardiovascular events (MACE), QTc prolongation, and discontinuation of study medication. We identified 5 RCTs with 773 participants who met the inclusion criteria. Escitalopram was not associated with significantly increased risk of MACE (risk ratio [RR] = 1.85; 95% confidence interval [CI] 0.80 to 4.26; *I*^2^ 0%; 5 RCTs; *n* = 773, moderate certainty of evidence), discontinuation of study medication (RR = 1.03; 95% CI 0.84–1.26; *I*^2^ 0%; 5 RCTs; *n* = 773, low certainty of evidence), and QTc prolongation (RR = 1.20; 95% CI 0.76–1.90; *I*^2^ 0%; 4 RCTs; *n* = 646, low certainty of evidence).

**Conclusion:**

Escitalopram does not significantly increase the risk of cardiovascular adverse reactions compared with placebo in patients with underlying cardiovascular disease. However, the presence of wide CIs and the limited number of included studies highlight the need for further studies with larger sample sizes to enhance the precision and reliability of these findings.

**Systematic review registration**: International Prospective Register of Systematic Reviews [CRD42022298181].

## 1. Introduction

Escitalopram is a selective serotonin reuptake inhibitor (SSRI) commonly prescribed for the treatment of psychiatric disorders such as major depressive, generalized anxiety, and obsessive-compulsive disorders (1). It was introduced in US market in 2002 and became available in a generic form in 2012 (1, 2). Its cost has significantly decreased since it became generic, resulting in increased global availability and improved cost-effectiveness. According to data from 2020, escitalopram is ranked the 15th most commonly prescribed medication in the United States (2).

Although escitalopram has been widely used and is generally considered safe, concerns have emerged regarding its potential for adverse cardiovascular reactions, specifically QT interval prolongation and risk of torsade de pointes. In 2011, the United Kingdom Medicines and Healthcare products Regulatory Agency issued a safety warnings highlighting the increased risk of QTc prolongation and cardiac outcomes associated with the use of escitalopram (3). An *in vitro* study demonstrated that escitalopram had the potential to delay ventricular repolarization, prolong QT intervals, and increase the risk of torsade de pointes by directly blocking potassium-hERG channels in cardiomyocytes (4). Additionally, a randomized controlled trial (RCT) conducted in 2016 indicated that the use of escitalopram might increase the risk of QTc prolongation in healthy human volunteers, leading to potentially fatal arrhythmias (5).

Furthermore, there have been reports indicating that depression and anxiety may be independently associated with poor prognoses in patients with cardiovascular diseases. Anxiety is associated with the onset and progression of cardiac disease and adverse cardiovascular outcomes, including mortality (6). Similarly, depression is associated with increased mortality, excess disability, higher health care costs, and reduced quality of life in patients with cardiovascular diseases (7). Despite the anticipated efficacy of escitalopram in the treatment of depression and anxiety in individuals with preexisting cardiovascular conditions, there are ongoing concerns regarding its potential adverse effects. Nevertheless, currently, there is a lack of comprehensive systematic reviews and meta-analyses specifically examining the risk-benefit balance assessment of escitalopram administration across all indications for patients with underlying cardiovascular diseases.

Thus, this systematic review and meta-analysis aimed to evaluate the risk-benefit balance of escitalopram treatment in patients with underlying cardiovascular disease. This assessment included quantification of the frequency and severity of adverse cardiovascular reactions and evaluation of the improvement of depressive or anxiety symptoms associated with escitalopram administration compared to those after using placebo in RCTs, particularly within the subgroup of patients with pre-existing cardiovascular diseases. By examining the available evidence, we aimed to provide a comprehensive and up-to-date evaluation of the cardiovascular safety profile and the impact on depressive or anxiety symptoms in individuals with underlying cardiovascular diseases who would benefit from escitalopram treatment. This evaluation may support clinical decision-making regarding the prescription of escitalopram for individuals experiencing anxiety or depressive symptoms within this specific patient population, potentially improving overall patient outcomes.

## 2. Methods

We followed the Preferred Reporting Items for Systematic Reviews and Meta-Analyses (PRISMA) guidelines (8). The protocol for this systematic review was registered in the International Prospective Register of Systematic Reviews (CRD42022298181).

### 2.1. Inclusion and exclusion criteria

Placebo-controlled RCTs involving participants diagnosed with underlying heart disease by healthcare professionals, regardless of underlying psychiatric disorders, were included. No restrictions were imposed on age, sex, ethnicity, or language. The intervention included escitalopram monotherapy, with no limitations on dose, frequency, or treatment duration. Patients taking cardiovascular medication such as antiplatelets, anticoagulants, and beta-blockers were included because they were necessary for the treatment of underlying cardiovascular disease. Interventions involving other antidepressants, antipsychotics, or electroconvulsive therapies were excluded.

### 2.2. Outcomes

The primary outcomes were major adverse cardiovascular events (MACE), QTc prolongation, and discontinuation of study medication. MACE was defined as a composite of all-cause mortality, myocardial infarction (MI), and percutaneous coronary intervention (9). The secondary outcomes were all-cause mortality, cardiac death, nonfatal MI, all-cause hospitalization, acute coronary syndrome, congestive heart failure, arrhythmia, chest pain, palpitation, hypertension, hypotension, syncope, depressive symptoms, and anxiety symptoms.

### 2.3. Search methods

We performed a comprehensive literature search on PubMed (02/12/2022), Cochrane Central Register of Controlled Trials (02/23/2022), Embase (12/16/2021), World Health Organization International Clinical Trials Registry Platform (02/23/2022), and ClinicalTrials.gov (02/23/2022) to identify relevant studies. We applied no search restrictions on the date, language, or publication status. The search strategy for each database is included in the Supplementary Table S1.

### 2.4. Selection of studies and data extraction

The search results that met the inclusion criteria were extracted from the databases and systematically managed using the review management software, Rayyan (10). Within the program, duplicate entries were identified and excluded. Two authors independently assessed the titles and abstracts of the identified references; if considered relevant by at least one author, they were included in the second screening phase. We obtained and reviewed the full texts of the included studies using the same criteria applied in the first screening process. We included studies for which both reviewers agreed upon. In cases of disagreement, we consulted a third author to make a final decision. We conducted data extraction using the same approach used in the second screening process. We contacted the authors of the studies to obtain additional data or clarifications, if necessary.

### 2.5. Measurement of outcomes

We used a Mantel-Haenszel random-effects model to estimate the risk ratios (RRs) with their corresponding 95% confidence intervals (CIs) for dichotomous variables. For continuous variables, we calculated the standardized mean differences (SMDs) with their corresponding 95% CIs using inverse variance weighting.

### 2.6. Assessment of risk of bias

**Figure 1:**
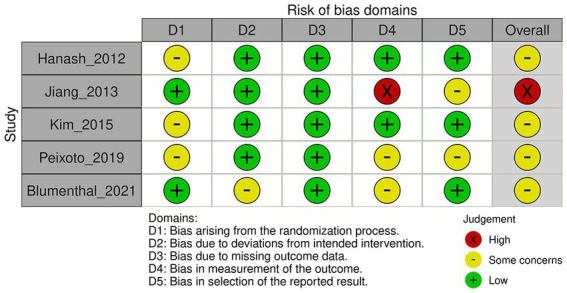
Risk of bias for major adverse cardiovascular events, discontinuation of study medication, and mortality according to version 2 of the Cochrane risk-of-bias tool.

**Figure 2:**
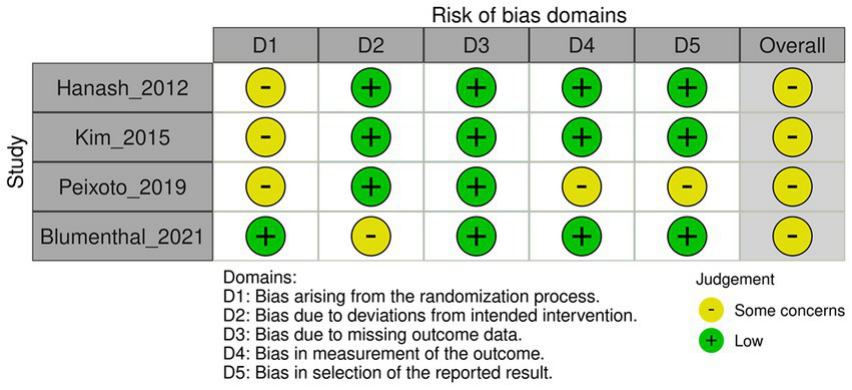
Risk of bias for QTc prolongation according to version 2 of the Cochrane risk-of-bias tool.

We assessed the risk of bias using version 2 of the Cochrane risk-of-bias tool for randomized trials (Figures 1, 2) (11). The risk-of-bias tool evaluates the following domains: bias arising from the randomization process, bias due to deviations from intended interventions, bias due to missing outcome data, bias in the measurement of outcomes, and bias in the selection of reported results. We assigned each domain a rating of low risk of bias, some concerns, or high risk of bias.

### 2.7. Analysis

Heterogeneity was assessed using the I^2^ statistic, with interpretation based on the Cochrane Handbook for Systematic Reviews of Interventions (0–40% may not be important; 30–60% may indicate moderate heterogeneity; 50–90% may indicate substantial heterogeneity; and 75–100% may indicate considerable heterogeneity) (12). If significant heterogeneity was observed, the source was further investigated. Specifically, sensitivity analysis was performed to assess the robustness of the results by excluding low-quality studies and studies with small sample sizes. Subgroup analysis was performed based on the proportion of female participants per trial, types of underlying cardiovascular diseases, and comorbid psychiatric disorders as potential moderators to examine potential heterogeneity and inconsistencies across the included studies based on participant characteristics. Statistical analyses were performed using the Review Manager software (version 5.4.1; Cochrane Collaboration).

The certainty of evidence for the primary outcomes was evaluated according to the Grading of Recommendations, Assessment, Development, and Evaluation rating (Supplementary Figure S12) (13).

## 3. Results

### 3.1. General description

**Figure 3:**
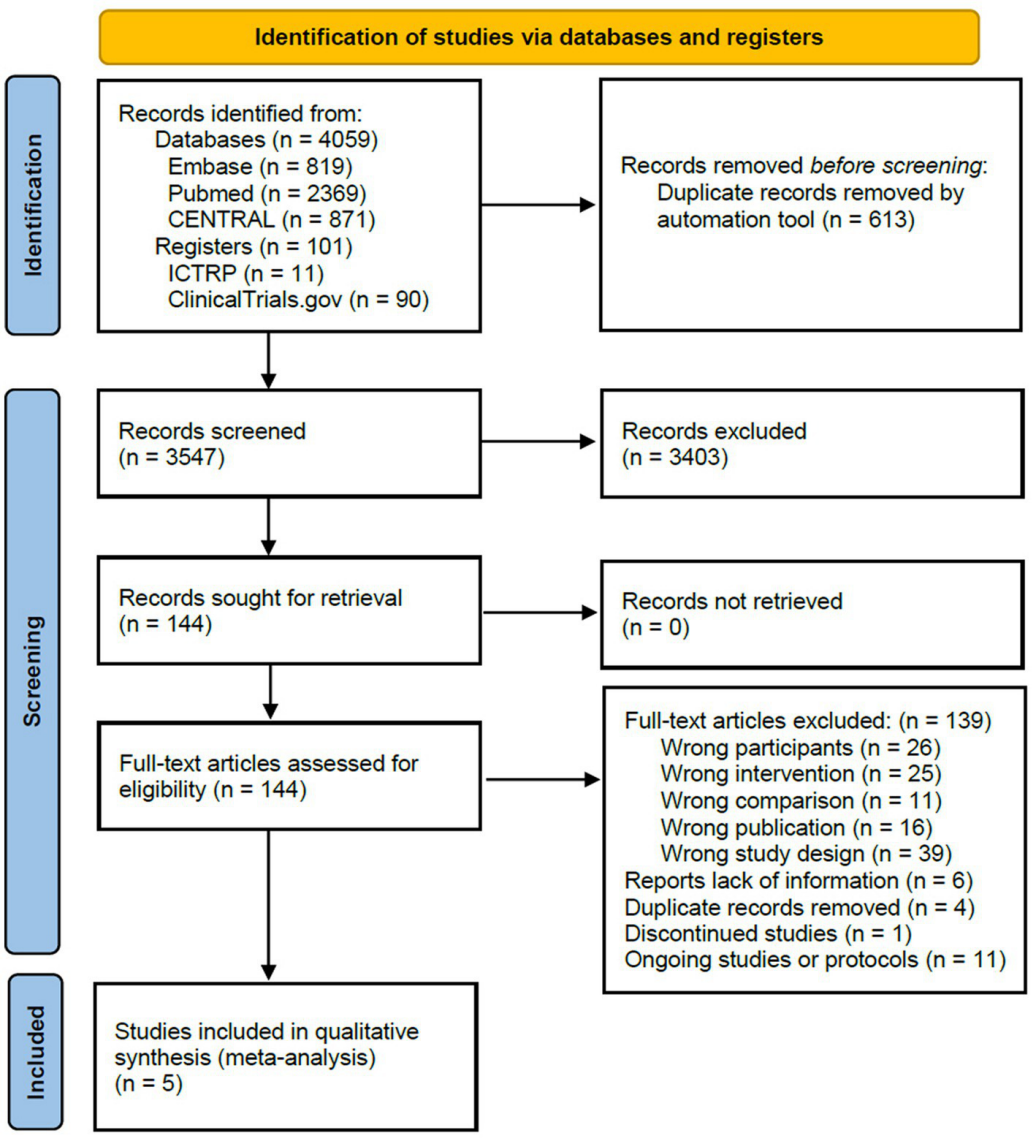
Preferred Reporting Items for Systematic Reviews and Meta-Analyses flow chart of included studies.

A total of 4,160 records were identified in the literature search process. After removing duplicates, two independent researchers reviewed 3,547 titles and abstracts. In the case of any disagreements between the researchers, a third reviewer was consulted for resolution. Following a thorough examination, a consensus was reached, resulting in 144 potentially relevant records. These 144 records were subjected to a comprehensive full-text review. Ultimately, five studies met the eligibility criteria and were included in this systematic review, which included 43 records of relevant data. The entire process is shown in Figure 3, the PRISMA flowchart.

### 3.2. Characteristics of included studies

Table 1 summarizes the key characteristics of the included studies. All studies met the inclusion criteria and were RCTs conducted using a parallel-group design. Among these studies, one was a three-arm trial (14), whereas the others were two-arm trials. The mean sample size per arm was 75, with a range of 15–151 participants. The studies were conducted in various regions, with two participants recruited from North America (15, 16), one from South America (14), one from Europe (17), and one from Asia (18). The proportion of female participants across the included studies ranged from 20.5 to 76.7%. The mean age of the participants varied from 57.2 to 64.8 years.

**Table 1:** Characteristics of the included studies.

Author; year; citation; country; study design	*N*	Age mean ± SD	% female	Underlying cardiovascular disease	Comorbid psychiatric disorders	Intervention	Comparator	Time point for follow-up (weeks)	Dose of escitalopram (mg/day)
Peixoto et al. (14); South America; RCT	30	57.2 ± 6.65	76.7	Hypertension	Depressive disorder	Escitalopram	Passive placebo	8	Flexible doses of 10–20
Jiang et al. (15); North America; RCT	127	64.0 ± 10.7	20.5	Ischemic heart disease	None	Escitalopram	Passive placebo	6	Began at 5, with titration to 20 in 3 weeks
Blumenthal et al. (16); North America; RCT	128	64.6 ± 9.6	28.9	Ischemic heart disease	Anxiety disorder	Escitalopram Exercise	Passive placebo	12	Flexible doses of 10–20
Hanash et al. (17); Europe; RCT	240	64.8 ± 12.1	36.9	Ischemic heart disease	None	Escitalopram	Passive placebo	52	10
Kim et al. (18); Asia; RCT	300	59.3 ± 10.8	40.0	Ischemic heart disease	Depressive disorder	Escitalopram	Passive placebo	24	Flexible doses of 5–20

Regarding baseline cardiologic conditions, the left ventricular ejection fraction at baseline was 61.3% (18). For heart failure risk stratification, two studies used the New York Heart Association (NYHA) classification at baseline, yielding the following results: NYHA class I was reported in 92.9% (15) and 57.7% (17), whereas NYHA class II or III was observed in 7.1% (15) and 42.3% (17) of the participants. One of the studies excluded patients with an ejection fraction of <30% or decompensated heart failure, pacemaker dependence, or resting blood pressure of >200/120 mmHg (16). Similarly, another study excluded patients with congestive heart failure, chronic renal failure and/or acute myocardial infarction, and secondary hypertension (14).

### 3.3. Risk of bias

#### 3.3.1. Bias arising from the randomization process

Three studies did not provide information on the allocation sequence concealment. However, two studies used block randomization (15) and sealed envelopes (16).

#### 3.3.2. Bias due to deviations from intended interventions

Four studies were conducted using a double-blind design, in which both participants and outcome assessors were blinded of the treatment assignments. The percentage of dropouts was balanced between arms. In one study, the intervention involved exercise (16), which meant that participants were aware of their treatment assignments. As a result, there is the potential for deviations to arise owing to trial contexts. However, notably, the percentage of dropouts was balanced among the arms, which suggests that these deviations do not have a significant effect on the study outcomes.

#### 3.3.3. Bias due to missing outcome data

In three of the studies, the percentage of missing outcome data exceeded 5%, but was balanced between each study arm (15, 17, 18). Although this suggests that the results are unlikely to be biased because of missing data, a high percentage of missing data may still affect the overall certainty of the evidence. Reasons for dropping out included lost to follow-up, withdrawing consent, experiencing adverse events, violating the study protocol, or lack of efficacy. In two other studies, the percentage of missing outcome data was <5% (14, 16).

#### 3.3.4. Bias in measurement of the outcomes

The primary outcome was assessed using the incidence of MACE, QTc prolongation, and discontinuation of study medication. In three of the included studies (14–16), blinding of the outcome assessor to the treatment allocation was compromised in relation to both MACE and discontinuation of study medication. Moreover, in one of these studies (15), it remained uncertain whether knowledge of the intervention influenced the obtained results. Furthermore, in one of the studies (14) assessing QTc prolongation, the outcome assessor was not blinded to the treatment allocation.

#### 3.3.5. Bias in selection of the reported result

All included studies were conducted in accordance with pre-specified protocols. In two studies, it was unclear whether the reported results were selected from multiple analyses of data or outcome measurements (14, 15).

### 3.4. Primary outcomes

#### 3.4.1. Major adverse cardiovascular events

**Figure 4:**
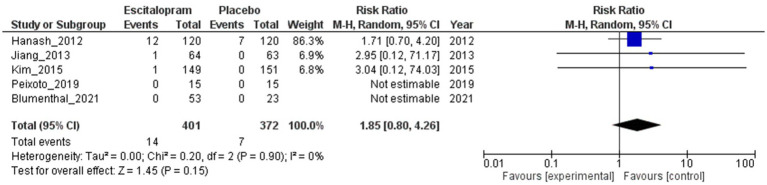
Escitalopram vs. placebo, major adverse cardiovascular events.

As illustrated in Figure 4, the risk of MACE was not significantly different between the escitalopram and placebo groups, as the 95% CI included 1 (RR = 1.85; 95% CI 0.80–4.26; 5 studies; 773 patients). Although an RR of 1.85 suggests a slightly higher risk of MACE in the escitalopram group, a CI that includes 1 indicates that the results are not statistically significant. The absence of heterogeneity among the studies reinforced the consistency of the findings (*I*^2^ = 0%; Tau^2^ = 0.00).

The sensitivity analysis, excluding studies with a high risk of bias (RR = 1.79; 95% CI 0.75–4.24; 4 studies; 646 patients) or small sample sizes (RR = 1.79; 95% CI 0.75–4.24; 2 studies; 540 patients), did not result in a significant change to the risk of MACE between the escitalopram and placebo groups (Supplementary Figures S1, S2). Similarly, subgroup analysis examining the effects based on the types of underlying cardiovascular diseases and comorbid psychiatric disorders did not yield significant alterations in the risk of MACE between the escitalopram and placebo groups (Supplementary Figures S3, S4). A subgroup analysis based on the proportion of female participants per trial could not be performed because of insufficient data availability.

#### 3.4.2. Discontinuation of study medication

**Figure 5:**
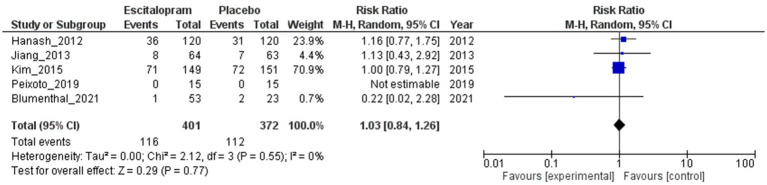
Escitalopram vs. placebo, discontinuation of study medication.

The results presented in Figure 5 indicated no statistically significant difference in the risk of discontinuation of study medication between the escitalopram- and placebo-treated groups. The RR was 1.03 (95% CI 0.84–1.26; 5 studies; 773 patients), indicating a slightly higher risk of discontinuation of study medication in the escitalopram group than in the placebo group, but this difference did not reach statistical significance as the 95% CI included the null value of 1.0. No heterogeneity was observed among the studies indicating consistency in the results (*I*^2^ = 0%; Tau^2^ = 0.00).

The sensitivity analysis, excluding studies with a high risk of bias (RR = 1.03; 95% CI 0.83–1.28; 4 studies; 646 patients) or small sample sizes (RR = 1.04; 95% CI 0.85–1.27; 2 studies; 540 patients), did not result in a significant change to the risk of discontinuation of study medication between the escitalopram and placebo groups (Supplementary Figures S5, S6). Similarly, a subgroup analysis examining the effects based on the types of underlying cardiovascular diseases and comorbid psychiatric disorders did not yield significant alterations in the risk of discontinuation of the study medication between the escitalopram and placebo groups (Supplementary Figures S7, S8). A subgroup analysis based on the proportion of female participants per trial could not be performed because of insufficient data availability.

#### 3.4.3. QTc prolongation

**Figure 6:**
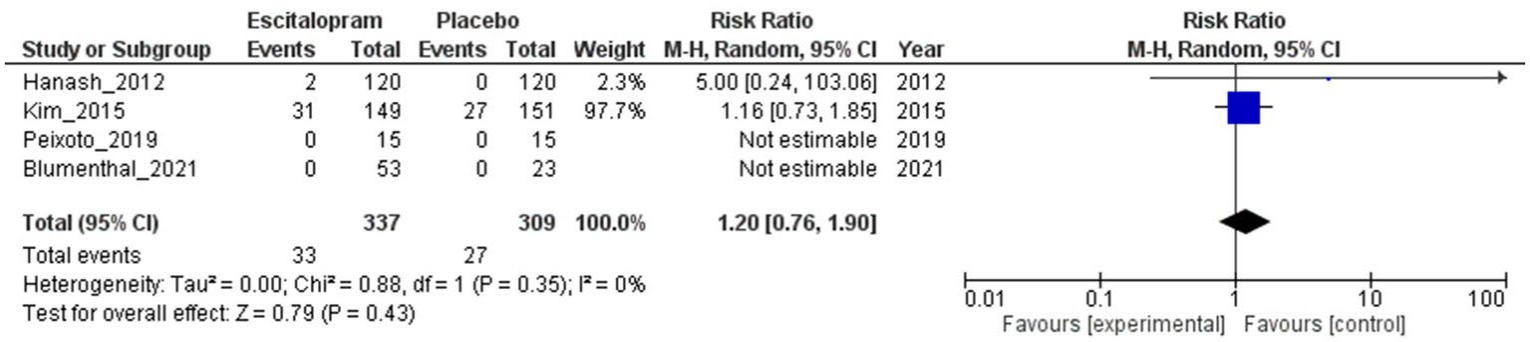
Escitalopram vs. placebo, QTc prolongation.

Figure 6 shows the results of the RR analysis of QTc prolongation in patients treated with escitalopram and placebo. The RR was 1.20 (95% CI 0.76–1.90; 4 studies; 646 patients), suggesting a slightly higher risk of QTc prolongation in the escitalopram group than in the placebo group, but the difference was not statistically significant as the 95% CI included the null value of 1.0. No heterogeneity was observed among the studies (*I*^2^ = 0%; Tau^2^ = 0.00), indicating consistency of the findings.

The sensitivity analysis, excluding studies with small sample sizes (RR = 1.20; 95% CI 0.76 to 1.90; 2 studies; 540 patients) did not result in a significant change to the risk of QTc prolongation between the escitalopram and placebo groups (Supplementary Figure S9). However, a sensitivity analysis excluding studies with a high risk of bias could not be performed because all included studies were found to have a moderate risk of bias. Subgroup analyses examining the effects of the types of underlying cardiovascular diseases and comorbid psychiatric disorders did not yield significant alterations in the risk of QTc prolongation between the escitalopram and placebo groups (Supplementary Figures S10, S11). A subgroup analysis based on the proportion of female participants per trial could not be performed because of insufficient data availability.

### 3.5. Secondary outcomes

#### 3.5.1. Mortality

**Figure 7:**
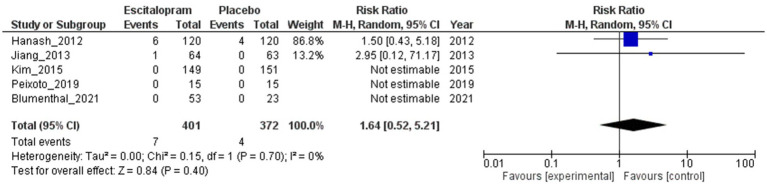
Escitalopram vs. placebo, mortality.

Figure 7 illustrates the outcomes of the RR analysis, which assessed mortality in patients administered escitalopram compared with those who received a placebo. The RR estimate was 1.64 (95% CI 0.52 to 5.21; 5 studies; 773 patients). This finding suggests a slightly elevated risk of mortality in the escitalopram group. However, this result was not statistically significant because the 95% CI included the null value of 1.0. The analysis revealed no heterogeneity among the studies (*I*^2^ = 0%; Tau^2^ = 0.00), indicating consistency in the results.

#### 3.5.2. Cardiac death

Two studies evaluated the incidence of cardiac death after treatment. Neither study reported cardiac death (15, 18).

#### 3.5.3. Nonfatal myocardial infarction

One study evaluated the occurrence of nonfatal MI after treatment. According to the study findings, no incidents of nonfatal MI were reported (16).

#### 3.5.4. Acute coronary syndrome

**Figure 8:**
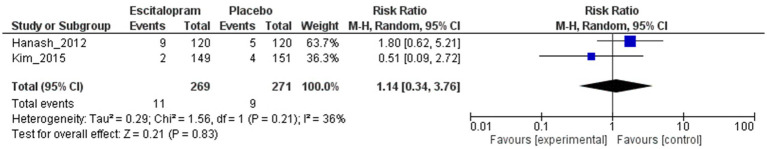
Escitalopram vs. placebo, acute coronary syndrome.

Two studies assessed the incidence of acute coronary syndrome after treatment, and RR analysis was performed to compare the incidence between patients who received escitalopram and a placebo. Figure 8 presents the results of the RR for acute coronary syndrome between patients treated with escitalopram and a placebo. The RR was 1.14 (95% CI 0.34 to 3.76; 2 studies; 540 patients), suggesting a slightly higher risk of acute coronary syndrome in the escitalopram group than in the placebo group, but the difference was not statistically significant as the 95% CI includes the null value of 1.0. Moderate heterogeneity was observed between the studies indicating inconsistency in the results (*I*^2^ = 36%; Tau^2^ = 0.29).

#### 3.5.5. Depressive symptoms

**Figure 9:**
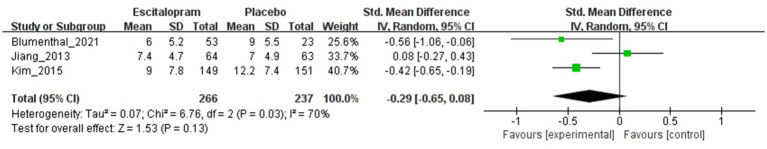
Escitalopram vs. placebo, post-treatment depressive symptoms.

Three studies assessed depressive symptoms after escitalopram treatment compared with placebo. Two studies used the Beck Depression Inventory (15, 16) and one study used the Montgomery-Asberg Depression Rating Scale (18) to assess the severity of depressive symptoms. An SMD analysis was performed to compare the severity of depressive symptoms between the two groups, and the results are shown in Figure 9. The analysis showed an estimated SMD of −0.29 (95% CI −0.65 to 0.08; 3 studies; 503 patients). However, substantial heterogeneity was observed among the studies indicating inconsistency in the results (*I*^2^ = 70%; Tau^2^ = 0.07).

#### 3.5.6. Anxiety symptoms

**Figure 10:**
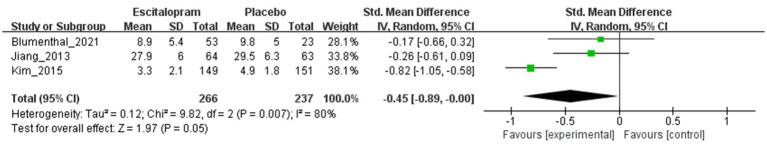
Escitalopram vs. placebo, post-treatment anxiety symptoms.

Three RCTs assessed anxiety symptoms after escitalopram treatment compared with placebo. The State–Trait Anxiety Inventory-State (15), Hamilton Anxiety Scale (16), and Hospital Anxiety and Depression Scale-Anxiety (18, 19) were used to assess the severity of the anxiety symptoms in each study. A meta-analysis using an SMD was performed to compare the severity of anxiety symptoms between the two groups, and the results are shown in Figure 10. The pooled analysis showed an estimated SMD of −0.45 (95% CI −0.89 to −0.00; 3 studies; 503 patients). Considerable heterogeneity was observed among the studies, indicating inconsistency in the results (*I*^2^ = 80%; Tau^2^ = 0.12).

## 4. Discussion

### 4.1. Summary of main results

In this systematic review, we identified five RCTs with 773 participants, that evaluated the risk of MACE and discontinuation of study medication associated with the use of escitalopram in patients with underlying heart disease. The moderate certainty of evidence indicated that the use of escitalopram did not significantly increase the risk of MACE compared with the placebo. However, caution is necessary because a wide CI implies considerable uncertainty in the estimate. Furthermore, the low certainty of evidence showed that the use of escitalopram did not significantly increase the risk of discontinuation of study medication compared with the placebo. These findings suggest that the rates of discontinuation of study medication are comparable between the two groups, implying that the use of escitalopram does not result in additional challenges regarding medication adherence.

Among these studies, four involving 646 participants specifically investigated the risk of QTc prolongation in patients with underlying heart diseases treated with escitalopram. The low certainty of evidence demonstrated that there was no significant increase in the risk of QTc prolongation when escitalopram was compared with the placebo. These results suggest that the risk of QTc prolongation is comparable between the two groups, indicating that escitalopram does not increase the risk of QTc prolongation in patients with underlying cardiovascular diseases. Heterogeneity was not observed in the primary outcomes.

Regarding secondary outcomes, escitalopram use in patients with underlying heart diseases did not significantly increase the risk of all-cause mortality or acute coronary syndrome. Two studies specifically evaluated the incidence of cardiac death and reported no cases of cardiac death. Additionally, a single study assessed the occurrence of nonfatal MI and found no reported incidents. These findings further support the notion that escitalopram does not increase the risk of adverse cardiovascular events leading to mortality.

Three studies assessed the effects of escitalopram treatment on depressive and anxiety symptoms compared with a placebo. In alignment with the findings from a previously published RCT in 2016 (20), conducted over an 18-month treatment period which reported no significant improvements in depressive symptoms when compared to a placebo among patients with chronic systolic heart failure and comorbid depression, our analysis revealed that escitalopram did not yield significant improvements in depressive symptoms when compared to a placebo among patients with underlying cardiovascular diseases. These consistent findings indicate the need for caution when considering the prescription of escitalopram within this specific patient population. However, a statistically significant trend toward reduced anxiety symptoms was identified in patients treated with escitalopram. These findings suggest that escitalopram may be effective in alleviating anxiety symptoms in patients with underlying cardiovascular diseases, highlighting the potential benefits of its use beyond cardiovascular safety. Nevertheless, the presence of substantial to considerable heterogeneity in post-treatment anxiety and depressive symptoms underscores the need to perform a meta-analysis that specifically focuses on individual psychiatric disorders to comprehensively evaluate the effects of escitalopram.

Based on the evidence obtained from this systematic review, the use of escitalopram in patients with underlying heart diseases does not significantly increase the risk of MACE, medication discontinuation, QTc prolongation, all-cause mortality, or acute coronary syndrome. The absence of observed heterogeneity in relation to the primary outcomes underscores the consistency and robustness of the findings and provides strong evidence for the cardiovascular safety of escitalopram in patients with underlying heart diseases. However, wide CIs in the estimates indicate a notable level of uncertainty, emphasizing the need for cautious interpretation of the results. Considering the relatively short follow-up duration of the included studies, which ranged from 6 to 52 weeks, additional studies with extended follow-up periods are required to improve evaluation, specifically concerning outcomes, such as MACE and overall mortality. Overall, our findings indicate the potential safety of escitalopram in this patient population; however, additional studies are required to validate and strengthen these conclusions.

### 4.2. Overall completeness and applicability of evidence

We performed a meta-analysis based on our preregistered protocol to avoid selective reporting bias and ensure transparency. A literature search identified 3,547 records, which were subsequently screened and assessed for eligibility. The final analysis was performed using data from 43 records of five studies. The included studies were conducted in different regions including Asia, Europe, North America, and South America. This geographical diversity enhances the generalizability of our findings, allowing for a more comprehensive understanding of the topic across various populations.

The risk-of-bias assessment within the included studies revealed that some lacked adequate information on allocation sequence concealment, and there was uncertainty regarding the blinding of outcome assessors. These factors may introduce a potential risk of bias, although the studies utilized a double-blind design, and the percentage of dropouts was balanced between treatment arms. Although the effect of these biases is likely to be minimal, they should be acknowledged when interpreting the results.

The analysis of the SMD for depressive and anxiety symptoms across the included studies highlighted significant heterogeneity (15, 16, 18). In the context of depressive symptoms, a sensitivity analysis was performed, which excluded Jiang et al. study (Supplementary Figure S13) (15). This exclusion resulted in a reduction in the observed heterogeneity (*I*^2^ = 0%; Tau^2^ = 0.00). and a statistically significant improvement in depressive symptoms (SMD −0.44; 95% CI −0.65 to −0.24; 2 studies; 376 patients). This analysis underscores the pivotal role of Jiang et al. study in driving the observed variability. Jiang et al. study reported no substantial improvement in depressive symptoms following escitalopram treatment, a contrast to the positive outcomes evident in both Kim et al. and Blumenthal et al. studies (16, 18), which indicated an amelioration of depressive symptoms after escitalopram administration. It is essential to acknowledge that Jiang et al. study had a shorter follow-up duration of 6 weeks than the other studies (12–24 weeks). Additionally, a distinguishing feature of Jiang et al. study was the absence of comorbid psychiatric disorders among participants, setting it apart from the other studies where participants exhibited comorbid depressive or anxiety disorders.

Shifting focus to the evaluation of anxiety symptoms, the inclusion of Kim et al. study introduced a source of heterogeneity into the analysis. The sensitivity analysis, involving the exclusion of Kim et al. study (18), yielded a reduction in the heterogeneity observed (*I*^2^ = 0%; Tau^2^ = 0.00) (Supplementary Figure S14). Kim et al. study stood out because of its larger participant sample size and extended follow-up duration of 24 weeks. Their study included participants diagnosed with major depressive disorder as a comorbid psychiatric disorder, in contrast to the other studies that included participants either without comorbidities or with anxiety disorders. Additionally, the geographical diversity between Kim et al. study, conducted in Asia, and the other studies conducted in North America, adds an additional layer of complexity to the analysis. The potential influence of cultural, social, and environmental factors on treatment outcomes warrants consideration.

In light of the findings, it is important to interpret the results with caution. Although the analysis did not reveal a statistically significant reduction in depressive symptoms associated with escitalopram administration, it did demonstrate a statistically significant reduction in anxiety symptoms linked to escitalopram use. However, the heterogeneity observed can be attributed to a combination of factors, including variations in the study duration, participant characteristics, comorbid psychiatric conditions, and geographical locations. These factors should be weighed when interpreting and extrapolating the outcomes. The existence of diverse conditions among the study populations may have contributed to the observed heterogeneity, potentially impacting the treatment effects unveiled in the meta-analysis. Therefore, a comprehensive understanding of the multifaceted nature of the study populations is crucial when interpreting the results.

This systematic review has some limitations. The number of included studies was relatively small, and the sample sizes varied. The longest follow-up period in the included studies was 52 weeks. The primary and secondary outcomes of this systematic review did not include the risk of abnormal bleeding events. However, SSRI therapy may increase the risk of abnormal bleeding events (21). Additionally, there is evidence suggesting the risk of bleeding with concurrent use of SSRI and aspirin (hazard ratio 1.42; 95% CI 1.08–1.87) compared with aspirin monotherapy, which is commonly prescribed in patients with underlying cardiovascular disease (22). Consequently, the long-term safety and efficacy of escitalopram, as well as the risk of abnormal bleeding events in patients with underlying cardiovascular diseases remain unclear. These limitations indicate the need for caution when interpreting our findings and highlight the importance of further studies.

Despite these limitations, we conducted a comprehensive synthesis of the available evidence to provide valuable insights into the safety and potential benefits of escitalopram in patients with underlying heart diseases. These findings suggest that the use of escitalopram within a 52-week timeframe is relatively safe for managing depressive and anxiety symptoms in this patient population.

To address these limitations and provide more robust evidence, future studies should focus on larger sample sizes, longer follow-up periods, and examination of the impact of concurrent anticoagulant or antiplatelet therapy. This would allow for a more comprehensive assessment of the safety profile, long-term effects, and potential benefits of escitalopram in patients with underlying heart diseases.

### 4.3. Quality of the evidence

All included studies were RCTs conducted using a parallel-group design; one study was a three-arm trial, whereas the others were two-arm trials. The studies were conducted in various regions, and the proportion of female participants across the included studies ranged from 20.5 to 76.7%. The mean age of the participants varied from 57.2 to 64.8 years.

Three studies did not provide information on allocation sequence concealment, and three of the studies had a percentage of missing outcome data exceeding 5%. The primary outcome was assessed using the incidence of MACE, QTc prolongation, and discontinuation of study medication. In three of the studies, the outcome assessor was not blinded to the treatment allocation. In two of the studies, it was unclear whether the reported results were selected from multiple analyses of data or outcome measurements.

Based on these findings, the risk of MACE was comparable between the groups, suggesting that escitalopram does not increase the risk of MACE in patients with underlying heart diseases. However, a wide CI implied considerable uncertainty in the estimate, and the certainty of the evidence was moderate because of the imprecision of the results (Supplementary Figure S12). Furthermore, the results suggested no statistically significant difference in the risk of discontinuation of study medication and QTc prolongation between the groups treated with escitalopram and placebo, with the certainty of evidence being low owing to the imprecision and indirectness of the results (Supplementary Figure S12).

Although the included studies were RCTs conducted using a parallel-group design and the risk of bias was assessed and reported, the certainty of evidence in this systematic review was low to moderate owing to the imprecision and indirectness of the results and limitations in the methodology and reporting of some of the included studies.

### 4.4. Potential biases in the review process

The strengths of this systematic review include the rigorous methodology employed, which adhered to current methodological standards. The review involved a comprehensive and independent search of electronic databases, with data extraction, analysis, and risk of bias assessment performed by two authors. To ensure objectivity, two authors independently interpreted the risk of bias domains and minimized bias in the assessment process. Additionally, the review aimed to minimize selection and language bias by not imposing restrictions on age, sex, ethnicity, language, sample size, or geographic region during the review process.

However, our study only included published studies despite our efforts to screen unpublished trials based on our pre-planned search strategy. Moreover, our search strategy did not cover gray literature. This limitation introduces the potential for publication bias, which can lead to an overrepresentation of studies demonstrating favorable outcomes for escitalopram. Consequently, this may have introduced bias and influenced the overall findings of our review.

Our review exclusively included studies written in English; however, we did not impose language restrictions to prevent language bias. This limitation raises the possibility that a language bias may have influenced our results. Despite conducting a comprehensive literature search, it is possible that relevant studies in other languages were inadvertently missed.

### 4.5. Agreements and disagreements with other studies or reviews

To the best of our knowledge, this is the first systematic review to assess the safety and effectiveness of escitalopram in patients with underlying cardiovascular diseases. Our findings are consistent with those of a previously published systematic review in 2022 (23), which included 11 RCTs involving participants diagnosed with stroke, who were randomly assigned to receive either escitalopram or a placebo. No significant differences were observed in the cardiovascular adverse effects between patients with stroke receiving escitalopram and those assigned to the placebo group in this study. Our systematic review incorporated five RCTs involving participants diagnosed with either ischemic heart disease or hypertension, further bolstering the evidence for the cardiovascular safety of escitalopram across different types of underlying cardiovascular diseases. In addition, our analysis demonstrated a statistically significant reduction in anxiety symptoms associated with escitalopram.

In a recent systematic Cochrane review published in 2021 (24), which included 30 RCTs involving participants diagnosed with coronary artery disease and comorbid depressive disorder and incorporated various types of interventions, such as psychotherapy and pharmacotherapy, the review failed to provide systematic evidence regarding the cardiovascular safety of escitalopram. This limitation arises from the inclusion of only one trial (9, 18, 19, 25–28) that directly compared escitalopram with a placebo. In contrast, our systematic review, which also included the aforementioned trials (9, 18, 19, 25–28), provides substantial evidence supporting the safety of escitalopram in patients with underlying cardiovascular disease. This is attributed to our analysis of cardiac death outcomes, which were derived from one additional RCTs (15), and our assessment of overall mortality, which was based on five RCTs (14–18).

In a network meta-analysis of 15 RCTs (29) involving participants diagnosed with depressive disorder, and comparing the cardiovascular safety of different SSRIs, escitalopram had significantly lower occurrence of cardiovascular side effects compared with paroxetine (odds ratio [OR] 0.37, 95% CI 0.14–0.77). Similarly, the findings indicated a lower risk of cardiovascular reactions to escitalopram compared with fluoxetine (OR 0.06, 95% CI 0.00 to 0.74). The authors concluded that treatment with escitalopram was associated with a lower risk of adverse cardiovascular reactions compared with other SSRIs. However, notably, the authors excluded patients with pre-existing cardiovascular diseases, which may have affected the evaluation of cardiovascular adverse reactions to escitalopram in patients with underlying cardiovascular diseases. Furthermore, the absence of a placebo group in this study made it challenging to quantify the risk of adverse reactions specifically attributed to escitalopram. To address these limitations, this systematic review employed a distinct approach. We exclusively included studies with designs involving placebo controls and patients diagnosed with underlying cardiovascular diseases. This deliberate methodology enabled us to accurately assess the risk of cardiovascular reactions relative to placebo in patients with underlying cardiovascular diseases. Consequently, our findings verified that escitalopram treatment was not associated with an increased risk of cardiovascular reactions in this patient population.

A prior comprehensive systematic review (30), which included six RCTs, six prospective studies, two cross-sectional studies, two pilot studies, one open-label study, and one secondary analysis involving participants diagnosed with heart failure and comorbid depressive disorder and incorporating various types of pharmacotherapy, including escitalopram, sertraline, and nefazodone, did not yield systematic evidence regarding the effectiveness and safety of escitalopram. This limitation arose from the fact that the findings were primarily based on one RCT (20) and two prospective studies (31, 32). The results of the RCT (20), which was excluded from our systematic review because of a lack of information about MACE and QTc prolongation, indicated that escitalopram treatment for a maximum of 24 months did not demonstrate a difference in depression severity compared with placebo, which aligns with the findings of our systematic review. However, our systematic review provided a more comprehensive and robust evaluation of the effect of escitalopram on depressive symptoms in this specific patient population. This is supported by our meta-analysis of these RCTs, which strengthens our conclusion regarding the effects of escitalopram on depressive symptoms.

In a prior meta-analysis (33), including two RCTs, two retrospective studies, and four prospective studies, participants diagnosed with heart failure were divided into antidepressant use and control groups for comparison. The findings revealed that the use of antidepressants was associated with an increased risk of all-cause mortality (RR 1.27; 95% CI 1.21–1.34) and cardiovascular mortality (RR 1.14; 95% CI 1.08–1.20). According to the 2021 European Society of Cardiology Guidelines on cardiovascular disease prevention in clinical practice, SSRIs are not recommended for patients with heart failure and major depression (34). Although we identified an RCT (20) that met our inclusion criteria and included patients with heart failure, it did not include the primary outcomes we targeted in our review, namely MACE and QT prolongation. Furthermore, the results of ongoing RCTs involving individuals with heart failure were not attainable for retrieval. Therefore, forthcoming RCTs focused on heart failure patients should incorporate assessments of MACE and QT prolongation. Caution is advised when applying our evidence to patients with heart failure, and further studies focusing specifically on this patient population are needed to make more precise recommendations.

In routine clinical practice, clinicians encounter a broad array of antidepressant options, necessitating substantial evidence to make optimal decisions for each patient. We anticipate that our findings will assume a pivotal role in guiding the prescription of escitalopram for individuals with underlying cardiovascular diseases experiencing anxiety or depressive symptoms. These results are expected to serve as a critical resource in shaping clinical guidelines and facilitating the collaborative decision-making process among patients, caregivers, and healthcare providers within everyday practice in this specific patient population. Future research endeavors should aim to expand upon our work by extending network meta-analysis techniques to integrate both aggregated and individual-patient data from clinical trials. This approach has the potential to predict personalized clinical outcomes, such as early treatment response or the occurrence of specific side effects.

## 5. Conclusion

Based on the available evidence from this systematic review, the use of escitalopram in patients with underlying heart diseases does not significantly increase the risk of MACE, discontinuation of study medication, or QTc prolongation compared with placebo. Additionally, escitalopram shows promise in reducing the severity of anxiety symptoms in this patient population. These results support the safe use of escitalopram as a treatment option for patients with underlying heart diseases. However, further studies are necessary to validate these findings and establish more robust evidence in this context. In each therapeutic attempt, the potential risks must be balanced against the benefits for the patient, considering the qualitative and quantitative effects of using a drug and the result to be expected if it is not administered.

## Data availability statement

The raw data supporting the conclusions of this article will be made available by the authors, without undue reservation.

## Author contributions

KS, HN, and HI conceptualized and designed the study. MK and YK developed the search strategies. KS, HN, HI, HA, SI, RS, TI, and MN independently screened the eligible studies. KS, HN, HI, HA, SI, and RS independently extracted the data from the included studies. KS performed the meta-analyses. HN and HI supervised all the phases of this review and resolved any disagreements to avoid errors. All authors contributed to the article and approved the submitted version.

## Funding

This study was supported by the Japan Society for the Promotion of Science KAKENHI (Grant Number: 20K1663800).

## Conflict of interest

HI has received honoraria for lectures from Otsuka Pharmaceutical and Viatris.

SI has received personal fees from Eisai, Janssen Pharmaceutical, Lundbeck, Meiji Seika Pharma, Otsuka Pharmaceutical, Sumitomo Pharma, and Takeda Pharmaceutical, and has received research/grant support from Eli Lilly.

IK has received honoraria from Boehringer Ingelheim, Eisai, Eli Lilly, Janssen Pharmaceutical, Meiji Seika Pharma, Mochida Pharmaceutical, Novartis Pharma, Otsuka Pharmaceutical, Shionogi, Sumitomo Pharma, Takeda Pharmaceutical, Tsumura, Viatris, and Yoshitomiyakuhin, and has received research/grant support from Asahi Kasei Pharma, Astellas, Daiichi Sankyo, Eisai, Eli Lilly, Mochida Pharmaceutical, Nihon Medi-Physics, Otsuka Pharmaceutical, Pfizer, Shionogi, Sumitomo Pharma, Takeda Pharmaceutical, and Tanabe Mitsubishi Pharma.

The remaining authors declare that the research was conducted in the absence of any commercial or financial relationships that could be construed as a potential conflict of interest.

## Publisher’s note

All claims expressed in this article are solely those of the authors and do not necessarily represent those of their affiliated organizations, or those of the publisher, the editors and the reviewers. Any product that may be evaluated in this article, or claim that may be made by its manufacturer, is not guaranteed or endorsed by the publisher.
