# Supplementary material for: Cardiovascular adverse reactions associated with escitalopram in patients with underlying cardiovascular diseases: a systematic review and meta-analysis
Source: Front Psychiatry. 2023 Sep 22;14:1248397. doi: 10.3389/fpsyt.2023.1248397 (PMC10556499; doi:10.3389/fpsyt.2023.1248397)
Supplement: Supplementary file 1 [file Data_Sheet_1.DOCX]

Supplementary Material

Cardiovascular adverse reactions associated with escitalopram in patients with underlying cardiovascular diseases: a systematic review and meta-analysis

Kenichi Kimura, Hisashi Narita^*^, Hissei Imai, Hisashi Akiyama, Shuhei Ishikawa, Ryo Sawagashira, Tomoyuki Isoyama, Mariko Nohara, Michiyo Kawamura, Yukari Kono, Takuya Saito, Ichiro Kusumi

*** Correspondence:** Hisashi Narita: aqualife99@hotmail.com

# Supplementary Tables

Supplementary Material Table S1. Search strategies

| Search strategy for PubMed |
| --- |
| #1 "heart diseases"[MeSH Terms] OR "Cardiovascular Physiological Phenomena"[Mesh] OR"cardio*"[Title/Abstract] OR "cardi*"[Title/Abstract] OR "heart*"[Title/Abstract] OR "coronary*"[Title/Abstract] OR "angina*"[Title/Abstract] OR "myocard*"[Title/Abstract] OR "ventricul*"[Title/Abstract] OR "atrioventricul*"[Title/Abstract] OR "pericard*"[Title/Abstract] OR "atrial*"[Title/Abstract] OR "endocardi*"[Title/Abstract] OR "arrhythmi*"[Title/Abstract] OR "thrombo*"[Title/Abstract] OR "tachy*"[Title/Abstract] OR "bradycardi*"[Title/Abstract] OR "fibrillat*"[Title/Abstract]  #2 "Citalopram"[Mesh] OR escitalopram[Title/Abstract] OR Lexapro[Title/Abstract] OR Mozarin[Title/Abstract] OR Aciprex[Title/Abstract] OR Depralin[Title/Abstract] OR Ecytara[Title/Abstract] OR Elicea[Title/Abstract] OR Nexpram[Title/Abstract] OR Pramatis[Title/Abstract] OR Betesda[Title/Abstract] OR Cipralex[Title/Abstract] OR Antidex[Title/Abstract] OR Asitaloks[Title/Abstract] OR benel[Title/Abstract] OR Celtium[Title/Abstract] OR Cilentra[Title/Abstract] OR "Cipra Pro"[Title/Abstract] OR Cironex[Title/Abstract] OR Citalam[Title/Abstract] OR Citalo[Title/Abstract] OR Citao[Title/Abstract] OR Citaplex[Title/Abstract] OR Citoles[Title/Abstract] OR Citram[Title/Abstract] OR Clomentin[Title/Abstract] OR Conjupram[Title/Abstract] OR Depram[Title/Abstract] OR Depresan[Title/Abstract] OR deprigen[Title/Abstract] OR deprilept[Title/Abstract] OR Depsit[Title/Abstract] OR Ecinil[Title/Abstract] OR Edpa[Title/Abstract] OR Elapram[Title/Abstract] OR Elxion[Title/Abstract] OR Epram[Title/Abstract] OR esciplex[Title/Abstract] OR escipram[Title/Abstract] OR escitactil[Title/Abstract] OR Escital[Title/Abstract] OR Escitalpro[Title/Abstract] OR escitasan[Title/Abstract] OR escitava[Title/Abstract] OR escitil[Title/Abstract] OR Escivex[Title/Abstract] OR Esidep[Title/Abstract] OR Esitalo[Title/Abstract] OR Eslopran[Title/Abstract] OR Esopam[Title/Abstract] OR Esoplex[Title/Abstract] OR Espran[Title/Abstract] OR Esram[Title/Abstract] OR Estimex[Title/Abstract] OR Esto[Title/Abstract] OR Estoram[Title/Abstract] OR Etalopro[Title/Abstract] OR Exodus[Title/Abstract] OR E-Zentius[Title/Abstract] OR Feliz S[Title/Abstract] OR Feliz S 10[Title/Abstract] OR Feliz S 20[Title/Abstract] OR Feliz-20[Title/Abstract] OR Ipran[Title/Abstract] OR Jovia[Title/Abstract] OR Leeyo[Title/Abstract] OR Lenuksyn[Title/Abstract] OR Lepax[Title/Abstract] OR Lexacure[Title/Abstract] OR Lexam[Title/Abstract] OR Lexamil[Title/Abstract] OR Lexapam[Title/Abstract] OR Lexapro[Title/Abstract] OR Lexapro Meltz[Title/Abstract] OR Lexatin[Title/Abstract] OR Lexcitam[Title/Abstract] OR Lexcitox[Title/Abstract] OR Lexdin[Title/Abstract] OR Losiram[Title/Abstract] OR Loxalate[Title/Abstract] OR L-Xapam[Title/Abstract] OR Meridian[Title/Abstract] OR Morcet[Title/Abstract] OR Neolexa[Title/Abstract] OR Neopra[Title/Abstract] OR Neopresol[Title/Abstract] OR Newpram[Title/Abstract] OR Nexcital[Title/Abstract] OR Nexito[Title/Abstract] OR Nodep[Title/Abstract] OR oroes[Title/Abstract] OR Oxapro[Title/Abstract] OR pralex[Title/Abstract] OR Pramokline[Title/Abstract] OR Purlex[Title/Abstract] OR Recita[Title/Abstract] OR Saropram[Title/Abstract] OR S-Celepra[Title/Abstract] OR Selectra[Title/Abstract] OR Seropam[Title/Abstract] OR Seroplex[Title/Abstract] OR servenon[Title/Abstract] OR Sipralexa[Title/Abstract] OR S-Oropram[Title/Abstract] OR Spador[Title/Abstract] OR symescital[Title/Abstract] OR Talopram[Title/Abstract] OR Zelax[Title/Abstract] OR Zytapram[Title/Abstract] OR Zytomil[Title/Abstract] OR Serotonin Uptake Inhibitors[MH] OR selective serotonin reuptake inhibitor*[Title/Abstract] OR SSRI*[Title/Abstract] OR "Serotonin Uptake Inhibitors" [Pharmacological Action] OR 5 Hydroxytryptamine Uptake Inhibitor*[Title/Abstract] OR 5 HT Uptake Inhibitor*[Title/Abstract]  #3 (randomized controlled trial[pt] OR controlled clinical trial[pt] OR randomized[Title/Abstract] OR placebo[Title/Abstract] OR drug therapy[sh] OR randomly[Title/Abstract] OR trial[Title/Abstract] OR groups[Title/Abstract] NOT (animals [mh] NOT humans [mh]))  #4 #1 AND #2 AND #3 |
| Search strategy for EMBASE |
| #1 cardiovascular function'/exp OR 'heart disease'/exp OR (cardi* OR heart* OR coronary* OR angina* OR myocard* OR ventricul* OR atrioventricul* OR pericard* OR atrial* OR endocardi* OR arrhythmi* OR thrombo* OR tachy* OR brady* OR fibrillat*):ti,ab,kw  #2 escitalopram/exp OR (escitalopram OR Aciprex OR Antidex OR Asitaloks OR benel OR Betesda OR Celtium OR Cilentra OR ’Cipra Pro' OR Cipralex OR Cironex OR Citalam OR Citalo OR Citao OR Citaplex OR Citoles OR Citram OR Clomentin OR Conjupram OR Depralin OR Depram OR Depresan OR deprigen OR deprilept OR Depsit OR E-Zentius OR Ecinil OR Ecytara OR Edpa OR Elapram OR Elicea OR Elxion OR Epram OR esciplex OR escipram OR escitactil OR Escital OR Escitalpro OR escitasan OR escitava OR escitil OR Escivex OR Esidep OR Esitalo OR Eslopran OR Esopam OR Esoplex OR Espran OR Esram OR Estimex OR Esto OR Estoram OR Etalopro OR Exodus OR Feliz S 10 OR Feliz S 20 OR Feliz S OR Feliz-20 OR Ipran OR Jovia OR L-Xapam OR Leeyo OR Lenuksyn OR Lepax OR Lexacure OR Lexam OR Lexamil OR Lexapam OR Lexapro Meltz OR Lexapro OR Lexapro OR Lexatin OR Lexcitam OR Lexcitox OR Lexdin OR Losiram OR Loxalate OR Meridian OR Morcet OR Mozarin OR Neolexa OR Neopra OR Neopresol OR Newpram OR Nexcital OR Nexito OR Nexpram OR Nodep OR oroes OR Oxapro OR pralex OR Pramatis OR Pramokline OR Purlex OR Recita OR S-Celepra OR S-Oropram OR Saropram OR Selectra OR Seropam OR Seroplex OR servenon OR Sipralexa OR Spador OR symescital OR Talopram OR Zelax OR Zytapram OR Zytomil OR Zytomil):ti,ab,kw OR 'Serotonin Uptake Inhibitor'/de OR ('selective serotonin reuptake inhibitor*' OR SSRI* OR '5 Hydroxytryptamine Uptake Inhibitor*' OR '5 HT Uptake Inhibitor*'):ti,ab,kw  #3 (‘randomized controlled trial’/de OR ‘controlled clinical trial’/de OR random*:ti,ab,tt OR ‘randomization’/de OR ‘intermethod comparison’/de OR placebo:ti,ab,tt OR (compare:ti,tt OR compared:ti,tt OR comparison:ti,tt) OR ((evaluated:ab OR evaluate:ab OR evaluating:ab OR assessed:ab OR assess:ab) AND (compare:ab OR compared:ab OR comparing:ab OR comparison:ab)) OR (open NEXT/1 label):ti,ab,tt OR ((double OR single OR doubly OR singly) NEXT/1 (blind OR blinded OR blindly)):ti,ab,tt OR ‘double blind procedure’/de OR (parallel NEXT/1 group*):ti,ab,tt OR (crossover:ti,ab,tt OR ‘cross over’:ti,ab,tt) OR ((assign* OR match OR matched OR allocation) NEAR/6 (alternate OR group OR groups OR intervention OR interventions OR patient OR patients OR subject OR subjects OR participant OR participants)):ti,ab,tt OR (assigned:ti,ab,tt OR allocated:ti,ab,tt) OR (controlled NEAR/8 (study OR design OR trial)):ti,ab,tt OR (volunteer:ti,ab,tt OR volunteers:ti,ab,tt) OR ‘human experiment’/de OR Trial:ti,tt) NOT ((((random* NEXT/1 sampl* NEAR/8 (‘cross section*’ OR questionnaire* OR survey OR surveys OR database or databases)):ti,ab,tt) NOT (‘comparative study’/de OR ‘controlled study’/de OR ‘randomised controlled’:ti,ab,tt OR ‘randomized controlled’:ti,ab,tt OR ‘randomly assigned’:ti,ab,tt)) OR (‘cross-sectional study’/de NOT (‘randomized controlled trial’/de OR ‘controlled clinical study’/de OR ‘controlled study’/de OR ‘randomised controlled’:ti,ab,tt OR ‘randomized controlled’:ti,ab,tt OR ‘control group’:ti,ab,tt OR ‘control groups’:ti,ab,tt)) OR (‘case control*’:ti,ab,tt AND random*:ti,ab,tt NOT (‘randomised controlled’:ti,ab,tt OR ‘randomized controlled’:ti,ab,tt)) OR (‘systematic review’:ti,tt NOT (trial:ti,tt OR study:ti,tt)) OR (nonrandom*:ti,ab,tt NOT random*:ti,ab,tt) OR ‘random field*’:ti,ab,tt OR (‘random cluster’ NEAR/4 sampl*):ti,ab,tt OR (review:ab AND review:it NOT trial:ti,tt) OR (‘we searched’:ab AND (review:ti,tt OR review:it)) OR ‘update review’:ab OR (databases NEAR/5 searched):ab OR ((rat:ti,tt OR rats:ti,tt OR mouse:ti,tt OR mice:ti,tt OR swine:ti,tt OR porcine:ti,tt OR murine:ti,tt OR sheep:ti,tt OR lambs:ti,tt OR pigs:ti,tt OR piglets:ti,tt OR rabbit:ti,tt OR rabbits:ti,tt OR cat:ti,tt OR cats:ti,tt OR dog:ti,tt OR dogs:ti,tt OR cattle:ti,tt OR bovine:ti,tt OR monkey:ti,tt OR monkeys:ti,tt OR trout:ti,tt OR marmoset*:ti,tt) AND ‘animal experiment’/de) OR (‘animal experiment’/de NOT (‘human experiment’/de OR ‘human’/de)))  #4 #1 AND #2 AND #3  #5 #4 NOT [medline]/lim |
| Search strategy for CENTRAL |
| #1 MeSH descriptor: [Heart Diseases] explode all trees  #2 MeSH descriptor: [Cardiovascular Physiological Phenomena] explode all trees  #3 (cardi*):ti,ab,kw  #4 (heart*):ti,ab,kw  #5 (coronary*):ti,ab,kw  #6 (angina*):ti,ab,kw  #7 (myocard*):ti,ab,kw  #8 (ventricul*):ti,ab,kw  #9 (atrioventricul*):ti,ab,kw  #10 (pericard*):ti,ab,kw  #11 (atrial*):ti,ab,kw  #12 (endocardi*):ti,ab,kw  #13 (arrhythmi*):ti,ab,kw  #14 (thrombo*):ti,ab,kw  #15 (tachy*):ti,ab,kw  #16 (brady*):ti,ab,kw  #17 (fibrillat*):ti,ab,kw  #18 #1 OR #2 OR #3 OR #4 OR #5 OR #6 OR #7 OR #8 OR #9 OR #10 OR #11 OR #12 OR #13 OR #14 OR #15 OR #16 OR #17  #19 MeSH descriptor: [Citalopram] explode all trees  #20 (escitalopram):ti,ab,kw  #21 (Aciprex):ti,ab,kw OR (Antidex):ti,ab,kw OR (Asitaloks):ti,ab,kw OR (benel):ti,ab,kw OR (Betesda):ti,ab,kw OR (Celtium):ti,ab,kw OR (Cilentra):ti,ab,kw OR ("Cipra Pro"):ti,ab,kw OR (Cipralex):ti,ab,kw OR (Cironex):ti,ab,kw OR (Citalam):ti,ab,kw OR (Citalo):ti,ab,kw OR (Citao):ti,ab,kw OR (Citaplex):ti,ab,kw OR (Citoles):ti,ab,kw OR (Citram):ti,ab,kw OR (Clomentin):ti,ab,kw OR (Conjupram):ti,ab,kw OR (Depralin):ti,ab,kw OR (Depram):ti,ab,kw OR (Depresan):ti,ab,kw OR (deprigen):ti,ab,kw OR (deprilept):ti,ab,kw OR (Depsit):ti,ab,kw OR (E-Zentius):ti,ab,kw OR (Ecinil):ti,ab,kw OR (Ecytara):ti,ab,kw OR (Edpa):ti,ab,kw OR (Elapram):ti,ab,kw OR (Elicea):ti,ab,kw OR (Elxion):ti,ab,kw OR (Epram):ti,ab,kw OR (esciplex):ti,ab,kw OR (escipram):ti,ab,kw OR (escitactil):ti,ab,kw OR (Escital):ti,ab,kw OR (Escitalpro):ti,ab,kw OR (escitasan):ti,ab,kw OR (escitava):ti,ab,kw OR (escitil):ti,ab,kw OR (Escivex):ti,ab,kw OR (Esidep):ti,ab,kw OR (Esitalo):ti,ab,kw OR (Eslopran):ti,ab,kw OR (Esopam):ti,ab,kw OR (Esoplex):ti,ab,kw OR (Espran):ti,ab,kw OR (Esram):ti,ab,kw OR (Estimex):ti,ab,kw OR (Esto):ti,ab,kw OR (Estoram):ti,ab,kw OR (Etalopro):ti,ab,kw OR (Exodus):ti,ab,kw OR (Feliz S 10):ti,ab,kw OR (Feliz S 20):ti,ab,kw OR (Feliz S):ti,ab,kw OR (Feliz-20):ti,ab,kw OR (Ipran):ti,ab,kw OR (Jovia):ti,ab,kw OR (L-Xapam):ti,ab,kw OR (Leeyo):ti,ab,kw OR (Lenuksyn):ti,ab,kw OR (Lepax):ti,ab,kw OR (Lexacure):ti,ab,kw OR (Lexam):ti,ab,kw OR (Lexamil):ti,ab,kw OR (Lexapam):ti,ab,kw OR (Lexapro Meltz):ti,ab,kw OR (Lexapro):ti,ab,kw OR (Lexapro):ti,ab,kw OR (Lexatin):ti,ab,kw OR (Lexcitam):ti,ab,kw OR (Lexcitox):ti,ab,kw OR (Lexdin):ti,ab,kw OR (Losiram):ti,ab,kw OR (Loxalate):ti,ab,kw OR (Meridian):ti,ab,kw OR (Morcet):ti,ab,kw OR (Mozarin):ti,ab,kw OR (Neolexa):ti,ab,kw OR (Neopra):ti,ab,kw OR (Neopresol):ti,ab,kw OR (Newpram):ti,ab,kw OR (Nexcital):ti,ab,kw OR (Nexito):ti,ab,kw OR (Nexpram):ti,ab,kw OR (Nodep):ti,ab,kw OR (oroes):ti,ab,kw OR (Oxapro):ti,ab,kw OR (pralex):ti,ab,kw OR (Pramatis):ti,ab,kw OR (Pramokline):ti,ab,kw OR (Purlex):ti,ab,kw OR (Recita):ti,ab,kw OR (S-Celepra):ti,ab,kw OR (S-Oropram):ti,ab,kw OR (Saropram):ti,ab,kw OR (Selectra):ti,ab,kw OR (Seropam):ti,ab,kw OR (Seroplex):ti,ab,kw OR (servenon):ti,ab,kw OR (Sipralexa):ti,ab,kw OR (Spador):ti,ab,kw OR (symescital):ti,ab,kw OR (Talopram):ti,ab,kw OR (Zelax):ti,ab,kw OR (Zytapram):ti,ab,kw OR (Zytomil):ti,ab,kw OR (Zytomil):ti,ab,kw  #22 MeSH descriptor: [Serotonin Uptake Inhibitors] explode all trees  #23 (selective serotonin reuptake inhibitor*):ti,ab,kw  #24 (SSRI*):ti,ab,kw  #25 (5 Hydroxytryptamine Uptake Inhibitor*):ti,ab,kw  #26 (5 HT Uptake Inhibitor*):ti,ab,kw  #27 #19 OR #20 OR #21 OR #22 OR #23 OR #24 OR #25 OR #26  #28 #18 AND #27 in Trials |
| Search strategy for ICTRP |
| #1 cardi* or heart* or coronary* or angina* or myocard* or ventricul* or atrioventricul* or pericard* or atrial* or endocardi* or arrhythmi* or thrombo* or tachy* or brady* or fibrillat*  #2 escitalopram OR Serotonin Uptake Inhibitor* OR SSRI*  #3 #1 AND #2 |
| Search strategy for CinicalTrials |
| Condition or disease: cardiovascular OR heart OR coronary OR angina OR myocardial OR ventricular OR atrioventricular OR pericardium OR atrial OR endocardial OR arrhythmia OR thrombo OR tachy OR brady OR fibrillation  Intervention/treatment: escitalopram OR "Serotonin Uptake Inhibitor" OR "Serotonin Uptake Inhibitors" OR SSRI OR SSRIs |

# Supplementary Figures

Supplementary Material Figure S1. Sensitivity analysis (major adverse cardiovascular events) based according to risk of bias (version 2 of the Cochrane risk-of-bias tool)


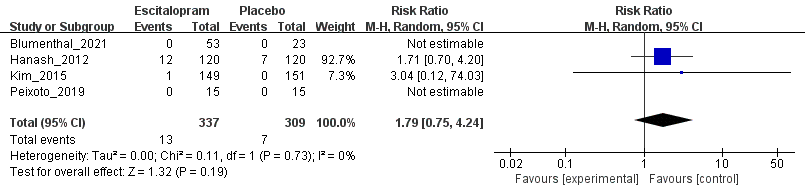


Supplementary Material Figure S2. Sensitivity analysis (major adverse cardiovascular events) according to sample size


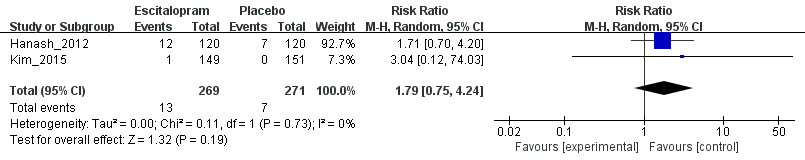


Supplementary Material Figure S3. Subgroup analysis (major adverse cardiovascular events) according to cardiovascular disease


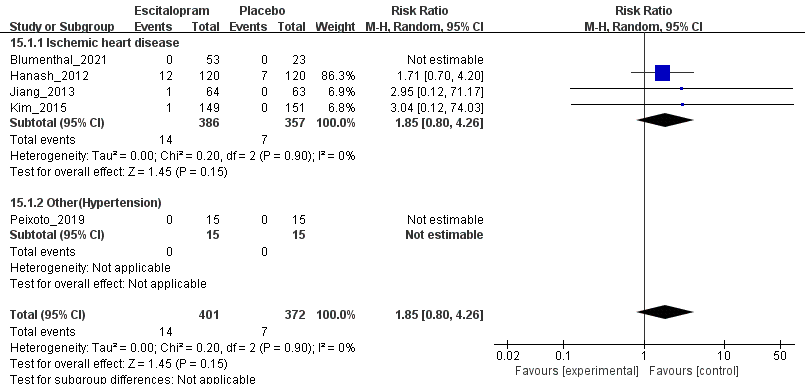


Supplementary Material Figure S4. Subgroup analysis (major adverse cardiovascular events) according to psychiatric disorder


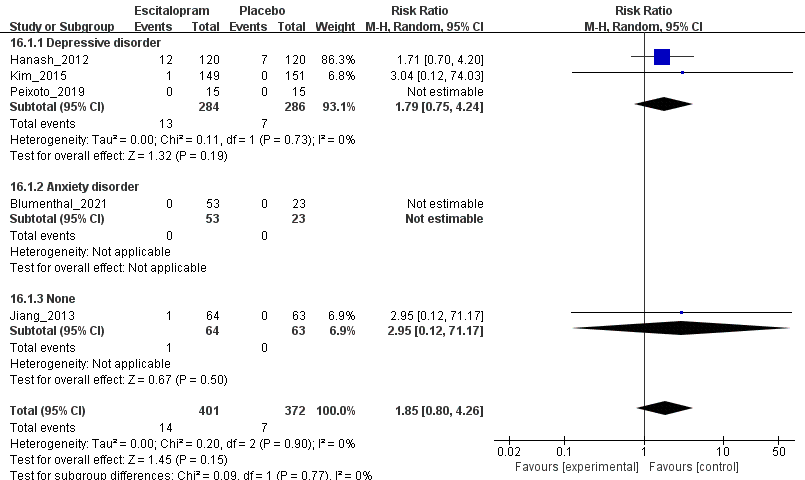


Supplementary Material Figure S5. Sensitivity analysis (discontinuation of the study medication) according to risk of bias (version 2 of the Cochrane risk-of-bias tool)


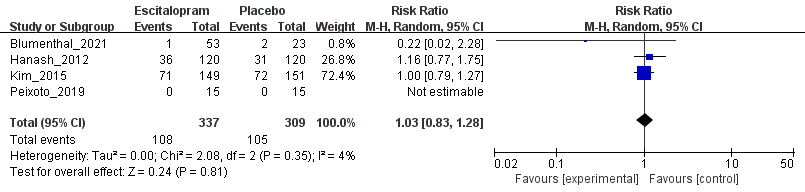


Supplementary Material Figure S6. Sensitivity analysis (discontinuation of the study medication) according to sample size


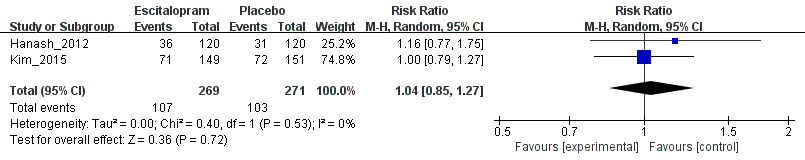


Supplementary Material Figure S7. Subgroup analysis (discontinuation of the study medication) according to cardiovascular disease


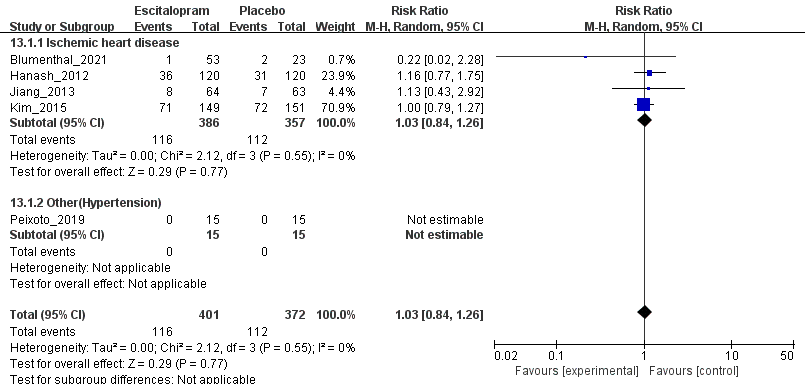


Supplementary Material Figure S8. Subgroup analysis (Discontinuation of the study medication) according to psychiatric disorder


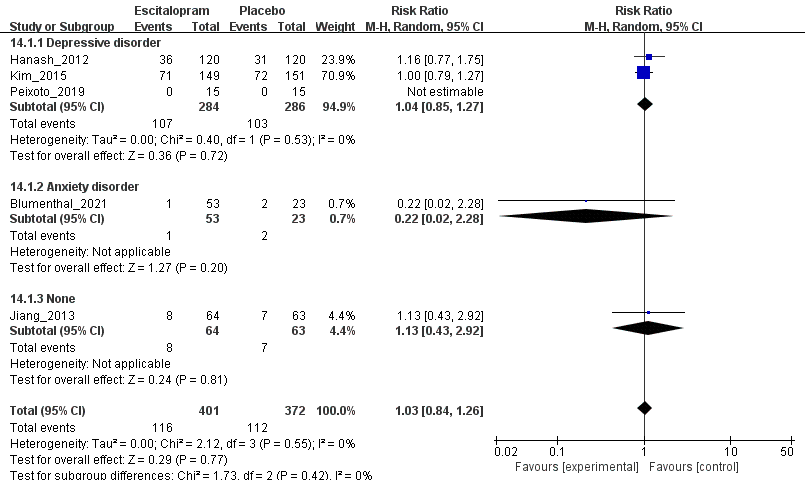


Supplementary Material Figure S9. Sensitivity analysis (QTc prolongation) according to sample size


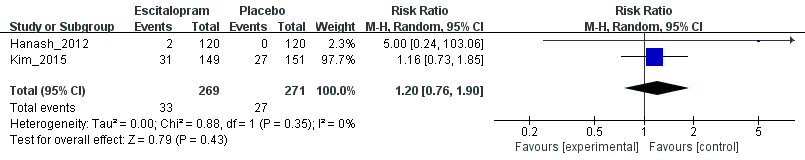


Supplementary Material Figure S10. Subgroup analysis (QTc prolongation) according to the cardiovascular disease


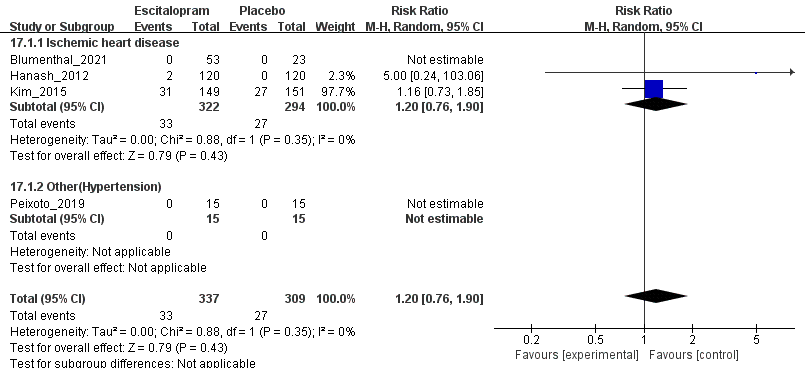


Supplementary Material Figure S11. Subgroup analysis (QTc prolongation) according to psychiatric disorder


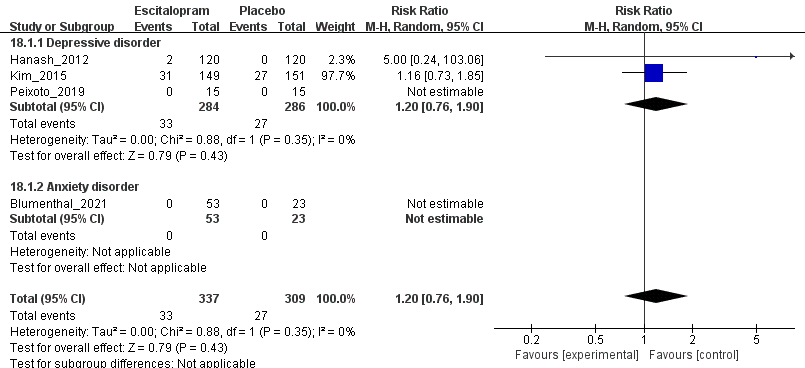


Supplementary Figure S12. Summary of findings based on the Grading of Recommendations, Assessment, Development, and Evaluation approach. Escitalopram compared with placebo in patients with underlying cardiovascular diseases


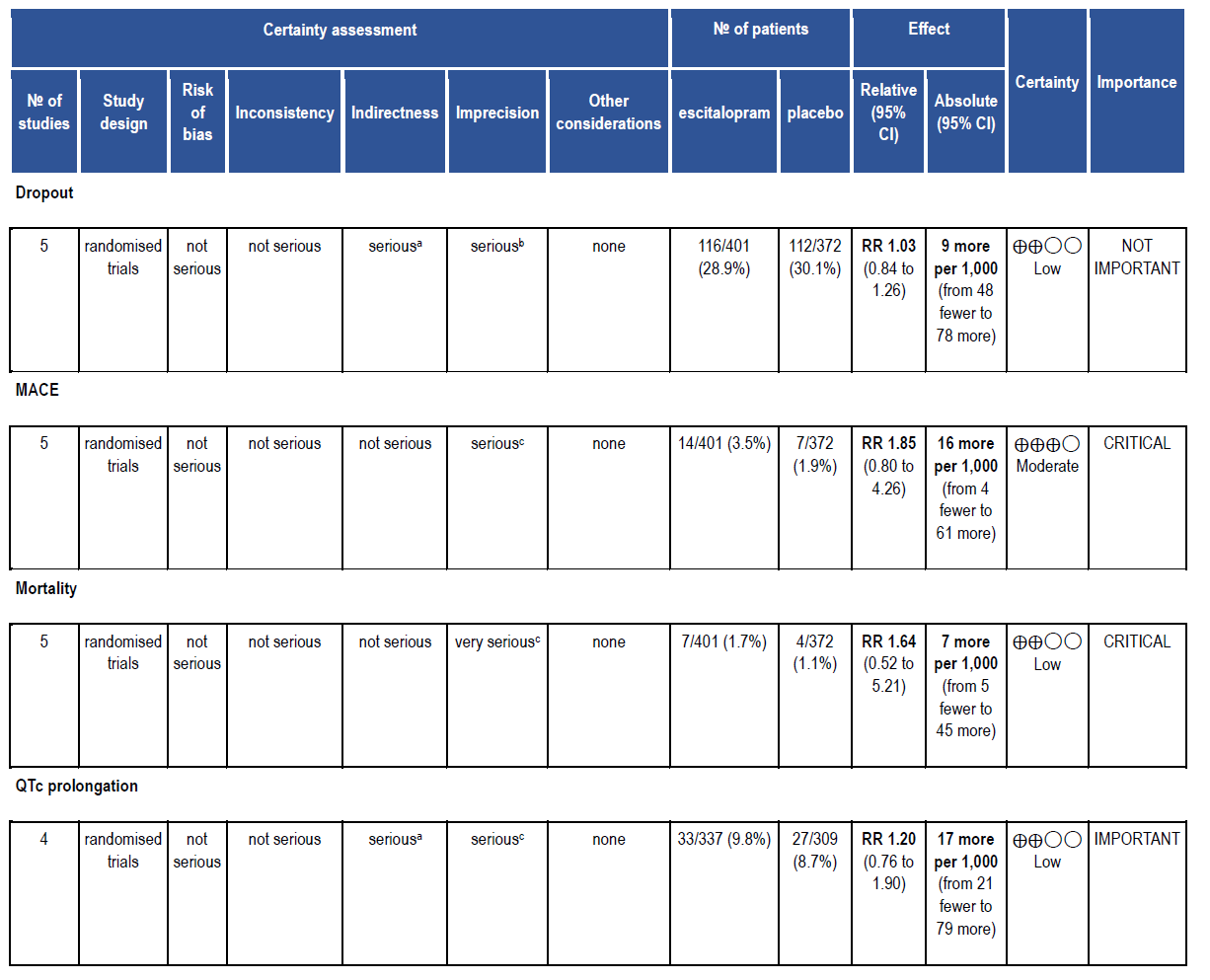


**GRADE levels of evidence**

High certainty: We are very confident that the true effect is close to that of the effect estimate.

Moderate certainty: We are moderately confident in the effect estimate: The true effect is likely to be close to the effect estimate, but there is a possibility that it is substantially different.

Low certainty: Our confidence in the effect estimate is limited: The true effect may be substantially different from the effect estimate.

Very low certainty: We have very little confidence in the effect estimate: The true effect is likely to be substantially different from the effect estimate.

**Explanations**

a. Surrogate outcomes

b. Confidence intervals cross null effect. Relatively few events (100~200).

c. Confidence intervals cross null effect. Few events (< 100).
